# Thyroid Hormone Signaling and Adult Neurogenesis in Mammals

**DOI:** 10.3389/fendo.2014.00062

**Published:** 2014-04-28

**Authors:** Sylvie Remaud, Jean-David Gothié, Ghislaine Morvan-Dubois, Barbara A. Demeneix

**Affiliations:** ^1^UMR CNRS 7221, Evolution des Régulations Endocriniennes, Département Régulations, Développement et Diversité Moléculaire, Muséum National d’Histoire Naturelle, Paris, France

**Keywords:** thyroid hormones, adult neurogenesis, brain functions, adult neural stem cells, plasticity, physiology

## Abstract

The vital roles of thyroid hormone in multiple aspects of perinatal brain development have been known for over a century. In the last decades, the molecular mechanisms underlying effects of thyroid hormone on proliferation, differentiation, migration, synaptogenesis, and myelination in the developing nervous system have been gradually dissected. However, recent data reveal that thyroid signaling influences neuronal development throughout life, from early embryogenesis to the neurogenesis in the adult brain. This review deals with the latter phase and analyses current knowledge on the role of T_3_, the active form of thyroid hormone, and its receptors in regulating neural stem cell function in the hippocampus and the subventricular zone, the two principal sites harboring neurogenesis in the adult mammalian brain. In particular, we discuss the critical roles of T_3_ and TRα1 in commitment to a neuronal phenotype, a process that entails the repression of a number of genes notably that encoding the pluripotency factor, *Sox2*. Furthermore, the question of the relevance of thyroid hormone control of adult neurogenesis is considered in the context of brain aging, cognitive decline, and neurodegenerative disease.

## Thyroid Hormones and Adult Brain Function

Thyroid hormones (THs) are vital for brain organization and function throughout life. In the developing mammalian embryo prior to instigation of fetal thyroid function maternal THs are required for optimal neurogenesis (1, 2). At all life stages, but particularly during perinatal growth, T_3_ is implicated in multiple processes including neurogenesis (cell cycle control and exit), synaptogenesis, migration, plasticity, and myelination (3). In adults, thyroid dysfunction correlates with neurological and behavioral disorders. Even if developmental hypothyroidism produces more deleterious, irreversible effects, adult hypothyroidism alters hippocampus function: memory impairment, anxiety, and depression-like symptoms in rodent models and humans (4, 5). In adults, the mechanisms underlying these cognitive problems are less well understood than during perinatal development. However, it is established that reduced neurogenesis, especially in the rodent hippocampus, due to either aging or stress, is associated with neurocognitive deficits such as anxiety, depression (6), and with neurodegenerative disease such as Alzheimer’s (7, 8). In mammals, including humans, the subgranular zone (SGZ) of the hippocampal dentate gyrus and the subventricular zone (SVZ) represent the two main neurogenic niches. These niches produce newborn neurons from neural stem cells (NSC) throughout life and so, contribute to brain plasticity during learning, memory, and recovery from brain damage (9). Many extrinsic and intrinsic signaling factors regulate different stages of adult neurogenesis (10), with TH signaling being well known to control NSC homeostasis [see below and (11–16)]. Understanding the mechanisms underlying T_3_ regulation of adult neurogenesis is crucial to develop treatments for neurocognitive disorders.

A rich literature links thyroid physiology and neurocognitive dysfunction in humans. Hypothyroidism is associated with mood instability and depression, dementia, memory impairment, and psychomotor problems (17). Most often, mood abnormalities reverse under T_4_-supplementation, but can persist after long-term hypothyroidism (18). The mechanisms implicated are unknown, although T_3_ levels affect serotoninergic and catecholaminergic signaling at multiple levels (19, 20), systems often targeted by anti-depressants. Further, in children and adolescents (21), as well as adults (22), hypothyroidism, and reduced memory function are associated with decreased hippocampal size, suggesting that TH deficiency causes structural alterations. Thus, it is plausible that neurogenesis in rodents, and depression or other psychiatric diseases associated with hypothyroidism in humans, may be related to reduced hippocampal neurogenesis.

However, the links between cognitive deficits and neurogenesis – “the neurogenic hypothesis of depression” – are still poorly understood. Even if there is evidence for adult neurogenesis in both SVZ (23) and SGZ (24) in humans, the contribution of adult neurogenesis to human brain function, and in particular to behavioral outputs, is still questioned, a point discussed in the next section.

However, there is increasing cellular and molecular understanding of the links between TH signaling and adult neurogenesis in rodents. Adult-onset hypothyroidism reduced the number of newborn neuroblasts in the dentate gyrus (14). Furthermore, in adult hypothyroid animals displaying depressive-like behavior, neurogenesis in the dentate gyrus is reduced and dendritic arborization is impaired. TH supplementation rescues these modifications (14).

## Thyroid Hormone Regulates Adult Neurogenesis

Neural stem cells in adult SGZ and SVZ slowly divide asymmetrically, giving rise to progenitors. In rodents, these highly proliferative progenitors generate neuroblasts that migrate and integrate into the pre-existing neuronal networks of the hippocampus and the olfactory bulb (OB). More recent findings highlight a third neurogenic niche within the adult rodent hypothalamus, a region regulating energy balance, food intake, and body weight (25, 26).

In humans, the functional role of adult neurogenesis is controversial (27–30). Both generation of new neuroblasts and their functional incorporation, especially in the OB, is still questioned. However, recent data showed that new neurons, probably produced from the adult SVZ, are observed in the human striatum, showing that adult human SVZ can contribute to neurogenesis at least in this region (31). A decrease of neuroblasts, expressing the neuronal precursor marker doublecortin (DCX), is observed continuously from the first year after birth, in the SVZ and SGZ (29, 30, 32, 33). However, a recent study shows that a subpopulation of hippocampal neurons is able to renew, supporting the concept that adult neurogenesis occurs in humans and could contribute to cognitive functions (24).

### SVZ and SGZ niches

Thyroid hormone signaling is one of the main pathways vital for adult neurogenesis. Recently, T_3_ was demonstrated to exert critical roles in cell proliferation and NSC commitment toward neuroblasts in both the rodent SVZ and SGZ *in vivo* (15, 16). T_3_ acts on transcription through nuclear receptors, Thyroid Hormone Receptors (TRs). In vertebrates, different isoforms derive from the *Thra* (TRα2 and TRα2) and *Thrb* (TRβ1 and TRβ2) genes. The adult hippocampus expresses TRα1, TRβ1, and β2 isoforms (16, 34), whereas only TRα1 is expressed in the adult mouse SVZ (13, 15).

T_3_ regulates adult neurogenesis at different steps (proliferation, survival, differentiation, and maturation). Hypothyroidism significantly reduces progenitor proliferation in the SVZ of adult mice, whereas a short T_3_ pulse restores mitotic activity to euthyroid levels (13). Similarly, using Ki67 as a proliferation marker and a BrdU incorporation protocol to measure cell proliferation limiting labeling of postmitotic cells, Montero-Pedrazuela et al. (14) demonstrated that hypothyroidism in adult rats, induces a decrease of proliferation (about 30%) in the adult SGZ that is reversed by T_4_ treatment. Furthermore, hypothyroidism does not affect cell survival. In contrast, two others studies shown that hypothyroidism had no observable effect on numbers of proliferative progenitors in the adult SGZ progenitor proliferation but their survival was reduced, suggesting a role of T_3_ on the postmitotic progenitors (11, 12). The reasons for these differences may reside in (i) methods for the induction of hypothyroidism (ii) and potential differences in BrdU protocols used in these studies that may or may not include postmitotic cells.

**Figure 1:**
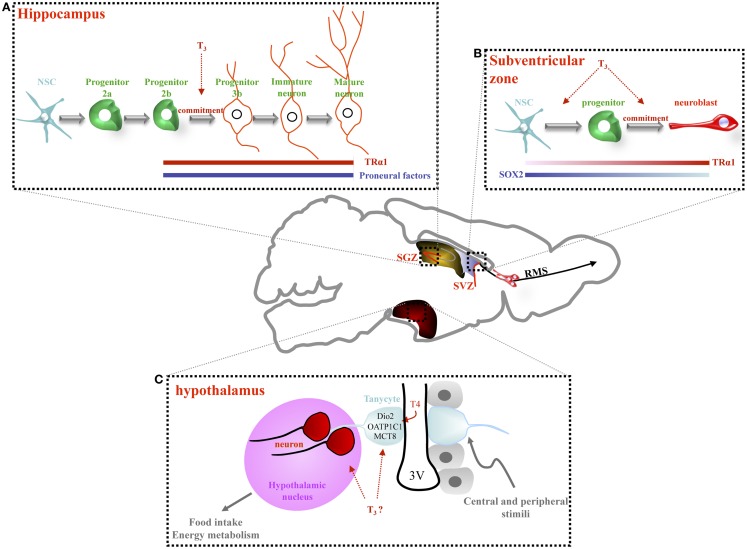
**Thyroid hormone signaling regulates adult neurogenesis in the hippocampus, the subventricular zone and, potentially, the hypothalamus**. **(A)** In the hippocampal niche (SGZ), NSC gives rise proliferating progenitors (2a) and then, more committed progenitors (2b) and post-mitotic neuroblasts (type 3). Type 3 progenitors give rise to immature and mature granule neurons. A role of T_3_, in concert with TRα1, has been observed in non-proliferating progenitors, from type 2b cells to mature granule cell neurons. Adult-onset hypothyroidism or TRα1 overexpression (TRα2^−/−^ mice) alters survival of post-mitotic neuroblasts, decreasing hippocampal neurogenesis. **(B)** In the adult SVZ, lining the lateral ventricle, three main cell types are located: NSCs that divide asymmetrically to give rise to proliferating progenitors. Progenitors divide rapidly producing neuroblasts that migrate along the rostral migratory stream (RMS) to the olfactory bulb (OB) where they differentiate into interneurons. SOX2 and TRα1 are inversely expressed within the SVZ: cells expressing high levels of TRα1 express low levels of SOX2 (neuroblasts). T_3_ is involved in both progenitor proliferation and determination. TRα1 overexpression in NSC and progenitors drives progenitor commitment toward a neuronal phenotype since cells overexpressing TRα1 are found in clusters entering the RMS. **(C)** In the adult hypothalamus, the third ventricle is lined by ependymal cells (in gray) interspersed with tanycytes (in blue). Some of these tanycytes are stem cells. They possess a long process that projects into hypothalamic nuclei (in pink). Some recent data support the idea that tanycytes are able to generate new neurons that migrate into adjacent hypothalamic nuclei. Tanycytes express many key actors of the TH pathway (Dio2, OATP1C1, and MCT8) thus, facilitating TH entry into the hypothalamus. These tanycytes could be considered as an “integrative platform” relaying central and peripheral signals to adapt adult neurogenesis to food intake and energy metabolism. A key role of TH in the regulation of adult hypothalamic neurogenesis is an exciting hypothesis.

In the SGZ, TRα1 has different effects on proliferation and differentiation (16, 35). First, progenitor proliferation is unaffected by TRα1 loss (TRα1^−/−^ mutant) or overexpression (TRα2^−/−^ mutant) (35). This finding correlates with the fact that TRα1 is not expressed in progenitors within the SGZ, but is highly expressed in post-mitotic progenitors corresponding to immature neurons (35). Second, neurogenesis is increased in TRα1^−/−^ mice, whereas in TRα2^−/−^ mice (overexpression of TRα1), decreased survival reduces numbers of post-mitotic neuroblasts (35). These studies suggest that in the SGZ, T_3_ acts at later steps than in the SVZ, in the post-mitotic progenitors (16, 35) (Figure 1A). Interestingly, the damaging effects of adult hypothyroidism on hippocampal neurogenesis are recapitulated in TRα2^−/−^ mice (35). The TRα2^−/−^ mutant, in which TRα1 is overexpressed due to the ablation of TRα2, exhibit a mixed hypo- and hyperthyroid phenotype: reduced levels of T_4_/T_3_ in serum, decreased growth rate and body weight, elevated heart rate suggesting that the increased TRα1 levels is associated with increased receptor effects (35, 36). In a hypothyroid context, TRα1 – in this mutant – acts as an aporeceptor due to limited T_3_ availability. How the role of TRα1 aporeceptor affects adult SVZ neurogenesis is unknown. Examining this possibility should identify new TRα1 targets (of both liganded and unliganded receptors) involved in regulating adult neurogenesis.

In the SVZ, although TRα1 is absent from NSCs, it appears in proliferative Dlx2+ progenitors and is high in DCX+ neuroblasts, suggesting that TRα1 favors NSC commitment toward a neuronal phenotype [(15), Figure 1B]. This hypothesis is bolstered by the observation that TRα1 gain of function *in vivo* generates migrating neuroblasts entering the rostral migratory stream. Inversely, shRNA-mediated TRα1 loss of function increases numbers of SVZ NSC/progenitors. Moreover, hypothyroidism also increases NSC/progenitor populations, a situation recapitulated in mutant *TRα/* mice (lacking all isoforms encoded by the *TR*α locus). In hypothyroidism, NSC/progenitors are blocked during interphase (13). Thus, absence of either TRα1 or T_3_ induces similar effects: increasing NSC and progenitors pools, while decreasing neuroblast numbers.

In the adult SVZ, T_3_, through TRα1, acts as a neurogenic switch by repressing a key gene involved in NSC pluripotency, *Sox2* (15) (Figure 1B). *In vivo* loss and gain of TRα1 function approaches demonstrated that *Sox2* is directly repressed by T_3_/TRα1 in progenitors. Moreover, the progenitor to neuroblast transition – governed by T_3_/TRα1 – may be reinforced by T_3_ repression of *CyclinD1* and *c-Myc*, involved in cell cycle progression (13, 15, 37). Thus, T_3_ could regulate adult SVZ homeostasis at two levels: (i) repression of a master gene involved in NSC pluripotency and (ii) repression of cell cycle regulators.

### TH signaling and hypothalamic neurogenesis?

Some authors consider that certain tanycytes (glial-like cells) in the ependymal layer are NSCs. An emerging idea is that these tanycytes are diet-responsive adult NSCs, linking food intake, body weight, and energy balance to neuronal plasticity [for reviews, see (25, 26)]. Interestingly, T_3_ is a strong regulator of energy metabolism at both peripheral and central, hypothalamic, levels (15). An exciting hypothesis is that T_3_ may regulate adult hypothalamic neurogenesis and thereby modulate plasticity of hypothalamic neuronal networks regulating energy balance. Many components of TH signaling are expressed in tanycytes in the rodent brain (D2, OATP1C1, MCT8, see Figure 1C) and in turn, tanycyte activity is critical to control of the hypothalamic/pituitary/thyroid (HPT) axis (38). How TH status and signaling affect adult hypothalamic neurogenesis in relation to feeding and energy balance is an important future research question.

## Control of T3 Availability during Adult Neurogenesis

Some T_3_ effects on stem cell biology can seem paradoxical, T_3_ enhancing both proliferation and differentiation and exerting different actions at successive steps of neural commitment. The biological outcome of TH signaling clearly relates to cellular context, notably, chromatin state and presence of ligand, TRs, and co-factors.

One hypothesis is that adult NSCs do not integrate T_3_ signaling until neural determination is underway, as TRα1 appears in neural progenitors, with the signal increasing in neuroblasts (15). In the TRα1:GFP knock-in mouse (39), expression of TRα1:GFP was not investigated closely in the SVZ. Although more data is needed on the kinetics of TR expression, a critical factor will be T_3_ availability, largely determined by deiodinases. Two deiodinases are expressed in the brain, the activating deiodinase 2 (or D2, encoded by *Dio2*) and the inactivating deiodinase 3 (or D3, encoded by *Dio3*). However, there is little published data on control of TH availability during neural determination and the little available is from *in vitro* systems. For instance, during *in vitro* neuronal differentiation of a human embryonal carcinoma stem cell line (NT2 cells derived from a teratocarcinoma), TRα1 and TRβ1 expression is down regulated, with TRα2 expression unchanged (40). T_3_ treatment induced stronger upregulation of *Dio3* in NT2 precursors than in differentiated cells.

Though hypothyroid brains show reduced NSC/precursor proliferation, no clear relationship between T_3_ availability and control of NSC cell cycle has yet been established. Interestingly, *Dio3* expression correlates with proliferative status in solid tumors (41). This finding fits with *in vitro* data [from Ref. (42)] where *Dio3* expression is high in early progenitors compared to human embryonic stem cells and neural progenitors. The biological significance of this finding in terms of NSC biology is hard to decipher. According to current data, local hypothyroidism favors maintenance of NSC/progenitor populations (13, 15) with T_3_ being a proliferation and neurogenic factor (15, 43). Similarly, expression of *Dio3* within the imprinted *dio3-dlk1* locus is associated with stemness (44). From an evolutionary point of view, the conservation of synteny in this locus among vertebrates seems to indicate that control of TH signaling is associated with stemness.

## TH Control of Adult Neurogenesis in the Aging Brain

Circulating TH levels decrease as a function of age in humans (45, 46) and rodents (47). In the aging human population, both increases and decreases in circulating TSH have been observed (48–51), suggesting reduced or impaired pituitary responses in elderly people. However, higher TSH is associated with greater longevity in numerous human cohorts [see for example: (52)]. Further, neurogenesis decreases with age (53–55). THs being vital for adult neurogenesis (13), it will be interesting to address the links between these phenomena during aging.

Among the numerous genes involved in adult neurogenesis, an increase in p16^INKA4^ (CDKN2a) has been causally related to neurogenic decline during aging (56). p16^INKA4^ can itself be inhibited by the synergistic action of Bmi1 and c-Myc (57, 58). Direct activation of *c-Myc* by T_3_ through a TRE was shown in Xenopus intestinal stem cells (59), whereas in adult SVZ T_3_ directly inhibits a *c-myc* reporter construct through an identified TRE (13). Thus, a potential indirect regulation of p16^INKA4^ by T_3_ could differ according to species, cell populations and function of developmental context.

### Decreasing circulating THs are associated with cognitive decline and neurodegeneration

Cognitive deficiency is frequently observed in the elderly humans and in aging rodents (60, 61). Marked effects are seen on learning and memory, processes that implicate neurogenesis in the dentate gyrus of the hippocampus (62, 63), a structure that diminishes with age and in many neurodegenerative pathologies (62, 64). TH treatment can improve cognitive performances in hypothyroid mice (8) and in humans (65), leading to speculation that cognitive deficiency can be causally linked to reduced TH signaling in aging. Despite declining neurogenesis with age, Yeung et al. recently demonstrated that 13-month-old mice still have the capacity to generate new neurons after a selective neuronal loss in the hippocampus, but without cognitive recovery (66). These results suggest that although some neurogenesis can still occur in aged mice, it might not be sufficient to compensate for neurodegeneration. TH facilitate repair after neurodegenerative lesions (67, 68). It is plausible that their decline is linked to decreased repair in neurodegenerative diseases of aging.

Mitochondrial biogenesis also reduces with aging (69), along with an increase in mitochondrial dysfunction (70). Thyroid signaling influences cellular metabolism and mitochondrial functions (71). Impaired thyroid signaling impacts mitochondrial respiration and hence reactive oxygen species (ROS) production, with either beneficial or damaging cellular effects (72). Since activity changes in mitochondrial respiration are linked to changes in cell proliferation rates (73), such as those occurring in the early phases of NSC differentiation, it can be postulated that mitochondrial dysfunctions impact neurogenesis, again linking reduced neurodegenerative repair capacity to decreased circulating T_3_/T_4_ levels. However, little is known about control of T_4_/T_3_ availability (deiodinase and TH transporter expression) during aging in the NSC niches, nor on the consequences of these modification for NSC metabolism, questions that it will be interesting to address.

Circadian rhythm perturbations also increase with age (74, 75). TSH (and to a lesser extent T_3_) levels display circadian rhythms (76–78), as does neurogenesis (79). Moreover, circadian clock-associated genes influence neuronal differentiation of adult NSC/progenitors (80). Two major circadian rhythm regulation genes, *Bmal1* and *Clock*, are cooperatively activated by *Sirt1* and *Pgc1a*, a function that changes with age (81). In turn, SIRT1 can act as a coactivator of TRβ (82) and is implicated in neurogenesis (83). Further, *Pgc1a* is directly regulated by T_3_ (84), and can itself modulate *Thra* expression (85). Some circadian clock-related genes are regulated by T_3_ (86). Thus, multiple arguments converge to suggest that impairments of circadian rhythm with age can be linked to changes in thyroid signaling, thereby impacting neurogenesis.

Induction of a chronic inflammatory state has been associated with aging (87, 88), and inflammation can significantly reduce neurogenesis (89–91). Brain inflammation is characterized by macrophages and microglia producing proinflammatory cytokines (TNFα, IL-1β, and IL-6) during prolonged inflammation. These same cytokines increase in the aging brain (92), and may enhance gliogenesis at the expense of neurogenesis (93–96). TNFα activates the p38 MAP kinase (MAPKp38) that triggers IL-1β production (97). As T_3_ can represses MAPKp38 activation by TNFα (98), reduced T_3_ dependent repression of proinflammatory cytokines with aging could negatively impact neurogenesis.

## Conclusion

Thyroid hormone is one of the few endocrine signals that exerts marked effects on both hippocampal and SVZ neurogenesis in adult mammalian brains. Although distinct differences are noted in expression of TRs and the consequences of their activation in these respective niches, it is well established that hypothyroidism adversely affects both populations. Given the frequency of thyroid disorders in the general population, notably in women and during aging, it is important to consider the consequences of these disorders on the incidence and severity of psychiatric and neurodegenerative disease.

## Conflict of Interest Statement

The authors declare that the research was conducted in the absence of any commercial or financial relationships that could be construed as a potential conflict of interest.

## References

[1] de Escobar GM Obregón MJ del Rey FE Maternal thyroid hormones early in pregnancy and fetal brain development. Best Pract Res Clin Endocrinol Metab (2004) 18:225–48 10.1016/j.beem.2004.03.012 15157838

[2] de Escobar GM Obregón MJ del Rey FE Iodine deficiency and brain development in the first half of pregnancy. Public Health Nutr (2007) 10:1554–70 10.1017/S1368980007360928 18053280

[3] Bernal J Thyroid hormone receptors in brain development and function. Nat Clin Pract Endocrinol Metab (2007) 3:249–59 10.1038/ncpendmet0424 17315033

[4] Dugbartey AT Neurocognitive aspects of hypothyroidism. Arch Intern Med (1998) 158:1413–8 10.1001/archinte.158.13.1413 9665349

[5] Fernández-Lamo I Montero-Pedrazuela A Delgado-García JM Guadaño-Ferraz A Gruart A Effects of thyroid hormone replacement on associative learning and hippocampal synaptic plasticity in adult hypothyroid rats. Eur J Neurosci (2009) 30(4):679–92 10.1111/j.1460-9568.2009.06862.x 19686470

[6] Mirescu C Gould E Stress and adult neurogenesis. Hippocampus (2006) 16:233–8 10.1002/hipo.20155 16411244

[7] Breteler MM van Duijn CM Chandra V Fratiglioni L Graves AB Heyman A Medical history and the risk of Alzheimer’s disease: a collaborative re-analysis of case-control studies. EURODEM Risk Factors Research Group. Int J Epidemiol (1991) 20(Suppl 2):S36–42 10.1093/ije/20.Supplement_2.S36 1833352

[8] Fu AL Zhou CY Chen X Thyroid hormone prevents cognitive deficit in a mouse model of Alzheimer’s disease. Neuropharmacology (2010) 58:722–9 10.1016/j.neuropharm.2009.12.020 20045708

[9] Ming G-L Song H Adult neurogenesis in the mammalian brain: significant answers and significant questions. Neuron (2011) 70:687–702 10.1016/j.neuron.2011.05.001 21609825 PMC3106107

[10] Suh H Deng W Gage FH Signaling in adult neurogenesis. Annu Rev Cell Dev Biol (2009) 25:253–75 10.1146/annurev.cellbio.042308.113256 19575663

[11] Ambrogini P Cuppini R Ferri P Mancini C Ciaroni S Voci A Thyroid hormones affect neurogenesis in the dentate gyrus of adult rat. Neuroendocrinology (2005) 81:244–53 10.1159/000087648 16113586

[12] Desouza LA Ladiwala U Daniel SM Agashe S Vaidya RA Vaidya VA Thyroid hormone regulates hippocampal neurogenesis in the adult rat brain. Mol Cell Neurosci (2005) 29:414–26 10.1016/j.mcn.2005.03.010 15950154

[13] Lemkine GF Raj A Alfama G Turque N Hassani Z Alegria-Prévot O Adult neural stem cell cycling in vivo requires thyroid hormone and its alpha receptor. FASEB J (2005) 19:863–5 10.1096/fj.04-2916fje 15728663

[14] Montero-Pedrazuela A Venero C Lavado-Autric R Fernández-Lamo I García-Verdugo JM Bernal J Modulation of adult hippocampal neurogenesis by thyroid hormones: implications in depressive-like behavior. Mol Psychiatry (2006) 11:361–71 10.1038/sj.mp.4001802 16446739

[15] López-Juárez A Remaud S Hassani Z Jolivet P Pierre Simons J Sontag T Thyroid hormone signaling acts as a neurogenic switch by repressing Sox2 in the adult neural stem cell niche. Cell Stem Cell (2012) 10:531–43 10.1016/j.stem.2012.04.008 22560077

[16] Kapoor R Desouza LA Nanavaty IN Kernie SG Vaidya VA Thyroid hormone accelerates the differentiation of adult hippocampal progenitors. J Neuroendocrinol (2012) 24:1259–71 10.1111/j.1365-2826.2012.02329.x 22497336

[17] Smith JW Evans AT Costall B Smythe JW Thyroid hormones, brain function and cognition: a brief review. Neurosci Biobehav Rev (2002) 26:45–60 10.1016/S0149-7634(01)00037-9 11835983

[18] Joffe RT Should thyroid replacement therapy be considered for patients with treatment-refractory depression? J Psychiatry Neurosci (2002) 27:80 11836979 PMC149799

[19] Henley WN Koehnle TJ Thyroid hormones and the treatment of depression: an examination of basic hormonal actions in the mature mammalian brain. Synapse (1997) 27:36–44 10.1002/(SICI)1098-2396(199709)27:1<36::AID-SYN4>3.0.CO;2-E 9268063

[20] Bauer M Heinz A Whybrow PC Thyroid hormones, serotonin and mood: of synergy and significance in the adult brain. Mol Psychiatry (2002) 7:140–56 10.1038/sj.mp.4000963 11840307

[21] Wheeler SM McAndrews MP Sheard ED Rovet J Visuospatial associative memory and hippocampal functioning in congenital hypothyroidism. J Int Neuropsychol Soc (2012) 18:49–56 10.1017/S1355617711001378 22114849

[22] Cooke G Mullally S Correia N O’Mara S Gibney J Hippocampal volume is decreased in adult-onset hypothyroidism. Thyroid (2013) 24:433–40 10.1089/thy.2013.0058 24205791

[23] Quiñones-Hinojosa A Sanai N Soriano-Navarro M Gonzalez-Perez O Mirzadeh Z Gil-Perotin S Cellular composition and cytoarchitecture of the adult human subventricular zone: a niche of neural stem cells. J Comp Neurol (2006) 494:415–34 10.1002/cne.20798 16320258

[24] Spalding KL Bergmann O Alkass K Bernard S Salehpour M Huttner HB Dynamics of hippocampal neurogenesis in adult humans. Cell (2013) 153:1219–27 10.1016/j.cell.2013.05.002 23746839 PMC4394608

[25] Bolborea M Dale N Hypothalamic tanycytes: potential roles in the control of feeding and energy balance. Trends Neurosci (2013) 36:91–100 10.1016/j.tins.2012.12.008 23332797 PMC3605593

[26] Cheng M-F Hypothalamic neurogenesis in the adult brain. Front Neuroendocrinol (2013) 34:167–78 10.1016/j.yfrne.2013.05.001 23684668

[27] Eriksson PS Perfilieva E Björk-Eriksson T Alborn AM Nordborg C Peterson DA Neurogenesis in the adult human hippocampus. Nat Med (1998) 4(11):1313–7 10.1038/3305 9809557

[28] Arellano JI Rakic P Neuroscience: gone with the wean. Nature (2011) 478:333–4 10.1038/478333a 22012389

[29] Sanai N Nguyen T Ihrie RA Mirzadeh Z Tsai H-H Wong M Corridors of migrating neurons in the human brain and their decline during infancy. Nature (2011) 478:382–6 10.1038/nature10487 21964341 PMC3197903

[30] Wang X Lui JH Kriegstein AR Orienting fate: spatial regulation of neurogenic divisions. Neuron (2011) 72:191–3 10.1016/j.neuron.2011.10.003 22017981 PMC3220621

[31] Ernst A Alkass K Bernard S Salehpour M Perl S Tisdale J Neurogenesis in the striatum of the adult human brain. Cell (2014) 156(5):1072–83 10.1016/j.cell.2014.01.044 24561062

[32] Göritz C Frisén J Neural stem cells and neurogenesis in the adult. Cell Stem Cell (2012) 10:657–9 10.1016/j.stem.2012.04.005 22704503

[33] Knoth R Singec I Ditter M Pantazis G Capetian P Meyer RP Murine features of neurogenesis in the human hippocampus across the lifespan from 0 to 100 years. PLoS One (2010) 5:e8809 10.1371/journal.pone.0008809 20126454 PMC2813284

[34] Kapoor R Ghosh H Nordstrom K Vennstrom B Vaidya VA Loss of thyroid hormone receptor β is associated with increased progenitor proliferation and NeuroD positive cell number in the adult hippocampus. Neurosci Lett (2011) 487:199–203 10.1016/j.neulet.2010.10.022 20959135 PMC4177097

[35] Kapoor R van Hogerlinden M Wallis K Ghosh H Nordstrom K Vennstrom B Unliganded thyroid hormone receptor alpha1 impairs adult hippocampal neurogenesis. FASEB J (2010) 24:4793–805 10.1096/fj.10-161802 20709911 PMC4177098

[36] Saltó C Kindblom JM Johansson C Wang Z Gullberg H Nordström K Ablation of TRalpha2 and a concomitant overexpression of Alpha1 yields a mixed hypo- and hyperthyroid phenotype in mice. Mol Endocrinol (2001) 15(12):2115–28 10.1210/mend.15.12.0750 11731613

[37] Hassani Z François J-C Alfama G Dubois GM Paris M Giovannangeli C A hybrid CMV-H1 construct improves efficiency of PEI-delivered shRNA in the mouse brain. Nucleic Acids Res (2007) 35:e65 10.1093/nar/gkm152 17426128 PMC1888798

[38] Fekete C Lechan RM Central regulation of hypothalamic-pituitary-thyroid axis under physiological and pathophysiological conditions. Endocr Rev (2013) 35:159–94 10.1210/er.2013-1087 24423980 PMC3963261

[39] Wallis K Susi D van Hogerlinden M Nordström K Mittag J Vennström B The thyroid hormone receptor Alpha1 protein is expressed in embryonic postmitotic neurons and persists in most adult neurons. Mol Endocrinol (2010) 24(10):1904–16 10.1210/me.2010-0175 20739404 PMC5417394

[40] Chan S McCabe CJ Visser TJ Franklyn JA Kilby MD Thyroid hormone responsiveness in N-Tera-2 cells. J Endocrinol (2003) 178:159–67 10.1677/joe.0.1780159 12844347

[41] Dentice M Marsili A Ambrosio R Guardiola O Sibilio A Paik J-H The FoxO3/type 2 deiodinase pathway is required for normal mouse myogenesis and muscle regeneration. J Clin Invest (2010) 120:4021–30 10.1172/JCI43670 20978344 PMC2964991

[42] Wu JQ Habegger L Noisa P Szekely A Qiu C Hutchison S Dynamic transcriptomes during neural differentiation of human embryonic stem cells revealed by short, long, and paired-end sequencing. Proc Natl Acad Sci U S A (2010) 107:5254–9 10.1073/pnas.0914114107 20194744 PMC2841935

[43] Chen C Zhou Z Zhong M Zhang Y Li M Zhang L Thyroid hormone promotes neuronal differentiation of embryonic neural stem cells by inhibiting STAT3 signaling through TRα1. Stem Cells Dev (2012) 21:2667–81 10.1089/scd.2012.0023 22468949 PMC3438880

[44] Liu L Luo G-Z Yang W Zhao X Zheng Q Lv Z Activation of the imprinted Dlk1-Dio3 region correlates with pluripotency levels of mouse stem cells. J Biol Chem (2010) 285:19483–90 10.1074/jbc.M110.131995 20382743 PMC2885227

[45] Chakraborti S Chakraborti T Mandal M Das S Batabyal SK Hypothalamic-pituitary-thyroid axis status of humans during development of ageing process. Clin Chim Acta (1999) 288:137–45 10.1016/S0009-8981(99)00061-3 10529465

[46] Hertoghe T The “multiple hormone deficiency” theory of aging: is human senescence caused mainly by multiple hormone deficiencies? Ann N Y Acad Sci (2005) 1057:448–65 10.1196/annals.1322.035 16399912

[47] Cao L Wang F Yang Q-G Jiang W Wang C Chen Y-P Reduced thyroid hormones with increased hippocampal SNAP-25 and Munc18-1 might involve cognitive impairment during aging. Behav Brain Res (2012) 229:131–7 10.1016/j.bbr.2012.01.014 22261019

[48] Boucai L Surks MI Reference limits of serum TSH and free T4 are significantly influenced by race and age in an urban outpatient medical practice. Clin Endocrinol (Oxf) (2009) 70:788–93 10.1111/j.1365-2265.2008.03390.x 18727705

[49] Hadlow NC Rothacker KM Wardrop R Brown SJ Lim EM Walsh JP The relationship between TSH and free T4 in a large population is complex and nonlinear and differs by age and sex. J Clin Endocrinol Metab (2013) 98:2936–43 10.1210/jc.2012-4223 23671314

[50] Surks MI Hollowell JG Age-specific distribution of serum thyrotropin and antithyroid antibodies in the US population: implications for the prevalence of subclinical hypothyroidism. J Clin Endocrinol Metab (2007) 92:4575–82 10.1210/jc.2007-1499 17911171

[51] Peeters RP Thyroid hormones and aging. Horm Athens Greece (2008) 7:28–35 10.14310/horm.2002.1111035 18359742

[52] Rozing MP Houwing-Duistermaat JJ Slagboom PE Beekman M Frölich M de Craen AJM Familial longevity is associated with decreased thyroid function. J Clin Endocrinol Metab (2010) 95:4979–84 10.1210/jc.2010-0875 20739380

[53] Enwere E Shingo T Gregg C Fujikawa H Ohta S Weiss S Aging results in reduced epidermal growth factor receptor signaling, diminished olfactory neurogenesis, and deficits in fine olfactory discrimination. J Neurosci (2004) 24:8354–65 10.1523/JNEUROSCI.2751-04.2004 15385618 PMC6729689

[54] Gould E Reeves AJ Fallah M Tanapat P Gross CG Fuchs E Hippocampal neurogenesis in adult Old World primates. Proc Natl Acad Sci U S A (1999) 96:5263–7 10.1073/pnas.96.9.5263 10220454 PMC21852

[55] Kuhn HG Dickinson-Anson H Gage FH Neurogenesis in the dentate gyrus of the adult rat: age-related decrease of neuronal progenitor proliferation. J Neurosci (1996) 16:2027–33 8604047 10.1523/JNEUROSCI.16-06-02027.1996 PMC6578509

[56] Molofsky AV Slutsky SG Joseph NM He S Pardal R Krishnamurthy J Increasing p16INK4a expression decreases forebrain progenitors and neurogenesis during ageing. Nature (2006) 443:448–52 10.1038/nature05091 16957738 PMC2586960

[57] Guney I Wu S Sedivy JM Reduced c-Myc signaling triggers telomere-independent senescence by regulating Bmi-1 and p16(INK4a). Proc Natl Acad Sci U S A (2006) 103:3645–50 10.1073/pnas.0600069103 16537449 PMC1450136

[58] Jacobs JJ Kieboom K Marino S DePinho RA van Lohuizen M The oncogene and Polycomb-group gene bmi-1 regulates cell proliferation and senescence through the ink4a locus. Nature (1999) 397:164–8 10.1038/16476 9923679

[59] Fujimoto K Matsuura K Hu-Wang E Lu R Shi Y-B Thyroid hormone activates protein arginine methyltransferase 1 expression by directly inducing c-Myc transcription during Xenopus intestinal stem cell development. J Biol Chem (2012) 287:10039–50 10.1074/jbc.M111.335661 22315222 PMC3323014

[60] Bach ME Barad M Son H Zhuo M Lu YF Shih R Age-related defects in spatial memory are correlated with defects in the late phase of hippocampal long-term potentiation in vitro and are attenuated by drugs that enhance the cAMP signaling pathway. Proc Natl Acad Sci U S A (1999) 96:5280–5 10.1073/pnas.96.9.5280 10220457 PMC21855

[61] Cao L Jiang W Wang F Yang Q-G Wang C Chen Y-P The reduced serum free triiodothyronine and increased dorsal hippocampal SNAP-25 and Munc18-1 had existed in middle-aged CD-1 mice with mild spatial cognitive impairment. Brain Res (2013) 1540:9–20 10.1016/j.brainres.2013.09.034 24134953

[62] Gould E Beylin A Tanapat P Reeves A Shors TJ Learning enhances adult neurogenesis in the hippocampal formation. Nat Neurosci (1999) 2:260–5 10.1038/6365 10195219

[63] van Praag H Schinder AF Christie BR Toni N Palmer TD Gage FH Functional neurogenesis in the adult hippocampus. Nature (2002) 415:1030–4 10.1038/4151030a 11875571 PMC9284568

[64] Zhao C Deng W Gage FH Mechanisms and functional implications of adult neurogenesis. Cell (2008) 132:645–60 10.1016/j.cell.2008.01.033 18295581

[65] Kramer CK von Mühlen D Kritz-Silverstein D Barrett-Connor E Treated hypothyroidism, cognitive function, and depressed mood in old age: the Rancho Bernardo Study. Eur J Endocrinol (2009) 161:917–21 10.1530/EJE-09-0606 19755406

[66] Yeung ST Myczek K Kang AP Chabrier MA Baglietto-Vargas D Laferla FM Impact of hippocampal neuronal ablation on neurogenesis and cognition in the aged brain. Neuroscience (2014) 259:214–22 10.1016/j.neuroscience.2013.11.054 24316470 PMC4438704

[67] Calzà L Fernandez M Giardino L Cellular approaches to central nervous system remyelination stimulation: thyroid hormone to promote myelin repair via endogenous stem and precursor cells. J Mol Endocrinol (2010) 44:13–23 10.1677/JME-09-0067 19578096

[68] Lin H-Y Davis FB Luidens MK Mousa SA Cao JH Zhou M Molecular basis for certain neuroprotective effects of thyroid hormone. Front Mol Neurosci (2011) 4:29 10.3389/fnmol.2011.00029 22016721 PMC3193027

[69] Derbré F Gomez-Cabrera MC Nascimento AL Sanchis-Gomar F Martinez-Bello VE Tresguerres JAF Age associated low mitochondrial biogenesis may be explained by lack of response of PGC-1α to exercise training. Age Dordr (2012) 34:669–79 10.1007/s11357-011-9264-y 21590341 PMC3337936

[70] Park CB Larsson N-G Mitochondrial DNA mutations in disease and aging. J Cell Biol (2011) 193:809–18 10.1083/jcb.201010024 21606204 PMC3105550

[71] Weitzel JM Iwen KA Coordination of mitochondrial biogenesis by thyroid hormone. Mol Cell Endocrinol (2011) 342:1–7 10.1016/j.mce.2011.05.009 21664416

[72] Long YC Tan TMC Inoue T Tang BL The biochemistry and cell biology of aging: metabolic regulation through mitochondrial signaling. Am J Physiol Endocrinol Metab (2014) 306:E581–91 10.1152/ajpendo.00665.2013 24452454

[73] Vander Heiden MG Cantley LC Thompson CB Understanding the Warburg effect: the metabolic requirements of cell proliferation. Science (2009) 324:1029–33 10.1126/science.1160809 19460998 PMC2849637

[74] Campos Costa I Nogueira Carvalho H Fernandes L Aging, circadian rhythms and depressive disorders: a review. Am J Neurodegener Dis (2013) 2:228–46 24319642 PMC3852564

[75] Froy O Circadian rhythms, aging, and life span in mammals. Physiology (Bethesda) (2011) 26:225–35 10.1152/physiol.00012.2011 21841071

[76] Bitman J Kahl S Wood DL Lefcourt AM Circadian and ultradian rhythms of plasma thyroid hormone concentrations in lactating dairy cows. Am J Physiol (1994) 266:R1797–803 8024031 10.1152/ajpregu.1994.266.6.R1797

[77] Gancedo B Alonso-Gómez AL de Pedro N Delgado MJ Alonso-Bedate M Changes in thyroid hormone concentrations and total contents through ontogeny in three anuran species: evidence for daily cycles. Gen Comp Endocrinol (1997) 107:240–50 10.1006/gcen.1997.6922 9245532

[78] Morris CJ Aeschbach D Scheer FAJL Circadian system, sleep and endocrinology. Mol Cell Endocrinol (2012) 349:91–104 10.1016/j.mce.2011.09.003 21939733 PMC3242827

[79] Bouchard-Cannon P Mendoza-Viveros L Yuen A Kærn M Cheng H-YM The circadian molecular clock regulates adult hippocampal neurogenesis by controlling the timing of cell-cycle entry and exit. Cell Rep (2013) 5:961–73 10.1016/j.celrep.2013.10.037 24268780

[80] Kimiwada T Sakurai M Ohashi H Aoki S Tominaga T Wada K Clock genes regulate neurogenic transcription factors, including NeuroD1, and the neuronal differentiation of adult neural stem/progenitor cells. Neurochem Int (2009) 54:277–85 10.1016/j.neuint.2008.12.005 19121353

[81] Chang H-C Guarente L SIRT1 mediates central circadian control in the SCN by a mechanism that decays with aging. Cell (2013) 153:1448–60 10.1016/j.cell.2013.05.027 23791176 PMC3748806

[82] Suh JH Sieglaff DH Zhang A Xia X Cvoro A Winnier GE SIRT1 is a direct coactivator of thyroid hormone receptor β1 with gene-specific actions. PLoS One (2013) 8:e70097 10.1371/journal.pone.0070097 23922917 PMC3724829

[83] Rafalski VA Ho PP Brett JO Ucar D Dugas JC Pollina EA Expansion of oligodendrocyte progenitor cells following SIRT1 inactivation in the adult brain. Nat Cell Biol (2013) 15:614–24 10.1038/ncb2735 23644469 PMC4026158

[84] Wulf A Harneit A Kröger M Kebenko M Wetzel MG Weitzel JM T3-mediated expression of PGC-1alpha via a far upstream located thyroid hormone response element. Mol Cell Endocrinol (2008) 287:90–5 10.1016/j.mce.2008.01.017 18336995

[85] Thijssen-Timmer DC Schiphorst MP-T Kwakkel J Emter R Kralli A Wiersinga WM PGC-1alpha regulates the isoform mRNA ratio of the alternatively spliced thyroid hormone receptor alpha transcript. J Mol Endocrinol (2006) 37:251–7 10.1677/jme.1.01914 17032743

[86] Diez D Grijota-Martinez C Agretti P De Marco G Tonacchera M Pinchera A Thyroid hormone action in the adult brain: gene expression profiling of the effects of single and multiple doses of triiodo-L-thyronine in the rat striatum. Endocrinology (2008) 149(8):3989–4000 10.1210/en.2008-0350 18467437

[87] Franceschi C Capri M Monti D Giunta S Olivieri F Sevini F Inflammaging and anti-inflammaging: a systemic perspective on aging and longevity emerged from studies in humans. Mech Ageing Dev (2007) 128:92–105 10.1016/j.mad.2006.11.016 17116321

[88] Strohacker K Breslin WL Carpenter KC McFarlin BK Aged mice have increased inflammatory monocyte concentration and altered expression of cell-surface functional receptors. J Biosci (2012) 37:55–62 10.1007/s12038-011-9169-z 22357203

[89] Butovsky O Ziv Y Schwartz A Landa G Talpalar AE Pluchino S Microglia activated by IL-4 or IFN-gamma differentially induce neurogenesis and oligodendrogenesis from adult stem/progenitor cells. Mol Cell Neurosci (2006) 31:149–60 10.1016/j.mcn.2005.10.006 16297637

[90] Ekdahl CT Claasen J-H Bonde S Kokaia Z Lindvall O Inflammation is detrimental for neurogenesis in adult brain. Proc Natl Acad Sci U S A (2003) 100:13632–7 10.1073/pnas.2234031100 14581618 PMC263865

[91] Monje ML Toda H Palmer TD Inflammatory blockade restores adult hippocampal neurogenesis. Science (2003) 302:1760–5 10.1126/science.1088417 14615545

[92] Terao A Apte-Deshpande A Dousman L Morairty S Eynon BP Kilduff TS Immune response gene expression increases in the aging murine hippocampus. J Neuroimmunol (2002) 132:99–112 10.1016/S0165-5728(02)00317-X 12417439

[93] Koo JW Duman RS IL-1beta is an essential mediator of the antineurogenic and anhedonic effects of stress. Proc Natl Acad Sci U S A (2008) 105:751–6 10.1073/pnas.0708092105 18178625 PMC2206608

[94] Lan X Chen Q Wang Y Jia B Sun L Zheng J TNF-α affects human cortical neural progenitor cell differentiation through the autocrine secretion of leukemia inhibitory factor. PLoS One (2012) 7:e50783 10.1371/journal.pone.0050783 23236394 PMC3517586

[95] Vallières L Campbell IL Gage FH Sawchenko PE Reduced hippocampal neurogenesis in adult transgenic mice with chronic astrocytic production of interleukin-6. J Neurosci (2002) 22:486–92 11784794 10.1523/JNEUROSCI.22-02-00486.2002 PMC6758670

[96] Zunszain PA Anacker C Cattaneo A Choudhury S Musaelyan K Myint AM Interleukin-1β: a new regulator of the kynurenine pathway affecting human hippocampal neurogenesis. Neuropsychopharmacology (2012) 37:939–49 10.1038/npp.2011.277 22071871 PMC3280640

[97] Kim SH Smith CJ Van Eldik LJ Importance of MAPK pathways for microglial pro-inflammatory cytokine IL-1 beta production. Neurobiol Aging (2004) 25:431–9 10.1016/S0197-4580(03)00126-X 15013563

[98] Lasa M Gil-Araujo B Palafox M Aranda A Thyroid hormone antagonizes tumor necrosis factor-alpha signaling in pituitary cells through the induction of dual specificity phosphatase 1. Mol Endocrinol (2010) 24:412–22 10.1210/me.2009-0298 20032197 PMC5428125

